# Could Alzheimer’s Disease Originate in the Periphery and If So How So?

**DOI:** 10.1007/s12035-018-1092-y

**Published:** 2018-04-29

**Authors:** Gerwyn Morris, Michael Berk, Michael Maes, Basant K. Puri

**Affiliations:** 1 IMPACT Strategic Research Centre, School of Medicine, Barwon Health, Deakin University, P.O. Box 291, Geelong, Victoria Australia; 2 Department of Psychiatry, Level 1 North, Main Block, Royal Melbourne Hospital, University of Melbourne, Parkville, Victoria Australia; 3 Florey Institute for Neuroscience and Mental Health, Kenneth Myer Building, University of Melbourne, 30 Royal Parade, Parkville, Victoria Australia; 4 Orygen, The National Centre of Excellence in Youth Mental Health, 35 Poplar Rd, Parkville, Victoria Australia; 5 Department of Psychiatry, Chulalongkorn University, Bangkok, Thailand; 6 Department of Medicine, Hammersmith Hospital, Imperial College London, London, UK

**Keywords:** Alzheimer’s disease, Gene expression, Inflammation, Microglia, Mitochondria, Molecular neurobiology

## Abstract

The classical amyloid cascade model for Alzheimer’s disease (AD) has been challenged by several findings. Here, an alternative molecular neurobiological model is proposed. It is shown that the presence of the *APOE* ε4 allele, altered miRNA expression and epigenetic dysregulation in the promoter region and exon 1 of *TREM2*, as well as *ANK1* hypermethylation and altered levels of histone post-translational methylation leading to increased transcription of *TNFA*, could variously explain increased levels of peripheral and central inflammation found in AD. In particular, as a result of increased activity of triggering receptor expressed on myeloid cells 2 (TREM-2), the presence of the apolipoprotein E4 (ApoE4) isoform, and changes in *ANK1* expression, with subsequent changes in miR-486 leading to altered levels of protein kinase B (Akt), mechanistic (previously mammalian) target of rapamycin (mTOR) and signal transducer and activator of transcription 3 (STAT3), all of which play major roles in microglial activation, proliferation and survival, there is activation of microglia, leading to the subsequent (further) production of cytokines, chemokines, nitric oxide, prostaglandins, reactive oxygen species, inducible nitric oxide synthase and cyclooxygenase-2, and other mediators of inflammation and neurotoxicity. These changes are associated with the development of amyloid and tau pathology, mitochondrial dysfunction (including impaired activity of the electron transport chain, depleted basal mitochondrial potential and oxidative damage to key tricarboxylic acid enzymes), synaptic dysfunction, altered glycogen synthase kinase-3 (GSK-3) activity, mTOR activation, impairment of autophagy, compromised ubiquitin-proteasome system, iron dyshomeostasis, changes in *APP* translation, amyloid plaque formation, tau hyperphosphorylation and neurofibrillary tangle formation.

## Introduction

**Fig. 1:**
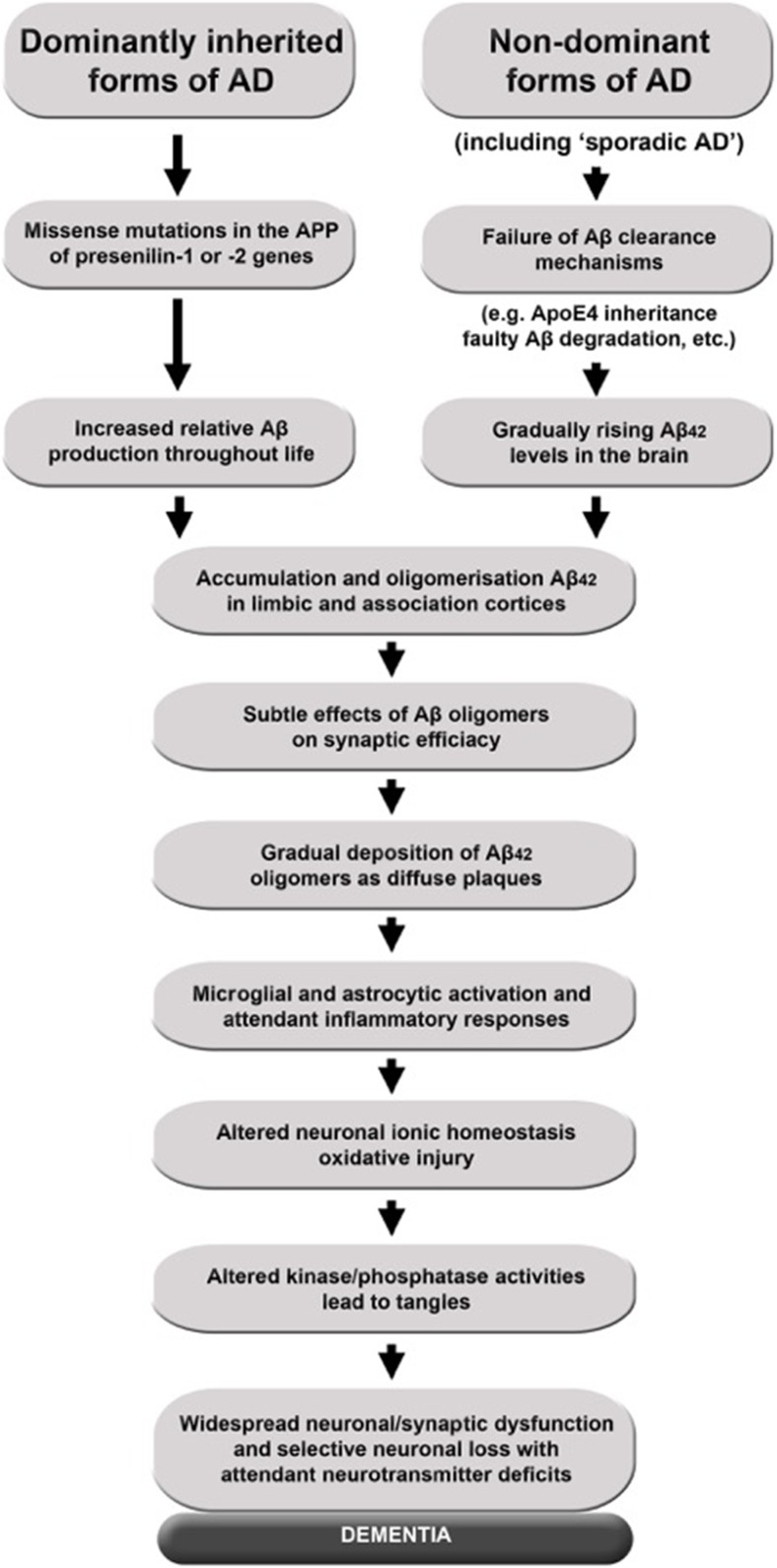
The amyloid hypothesis. According to the current ‘amyloid cascade’ model of disease causation, Aβ overproduction stems from the disruption of homeostatic mechanisms that regulate the proteolytic cleavage of APP under physiological conditions. This model proposes that age-related, genetic, epigenetic and environmental factors collude to provoke a metabolic shift favouring the processing of APP by BACE1 and the intramembranous γ-secretase complex composed in part by presenilin-1 or presenilin-2, while simultaneously inhibiting the physiological, secretory pathway via α-secretase, which releases soluble APPα which precludes generation of Aβ. The net result is to enhance the production of the putatively neurotoxic Aβ_42_ monomer at the expense of the putatively neuroprotective Aβ_40_. The current version of the amyloid hypothesis claims that Aβ_42_ accumulation into soluble oligomers is the primary driver of neuropathology, although the data allow for an independent or synergistic role for insoluble fibrils

**Fig. 2:**
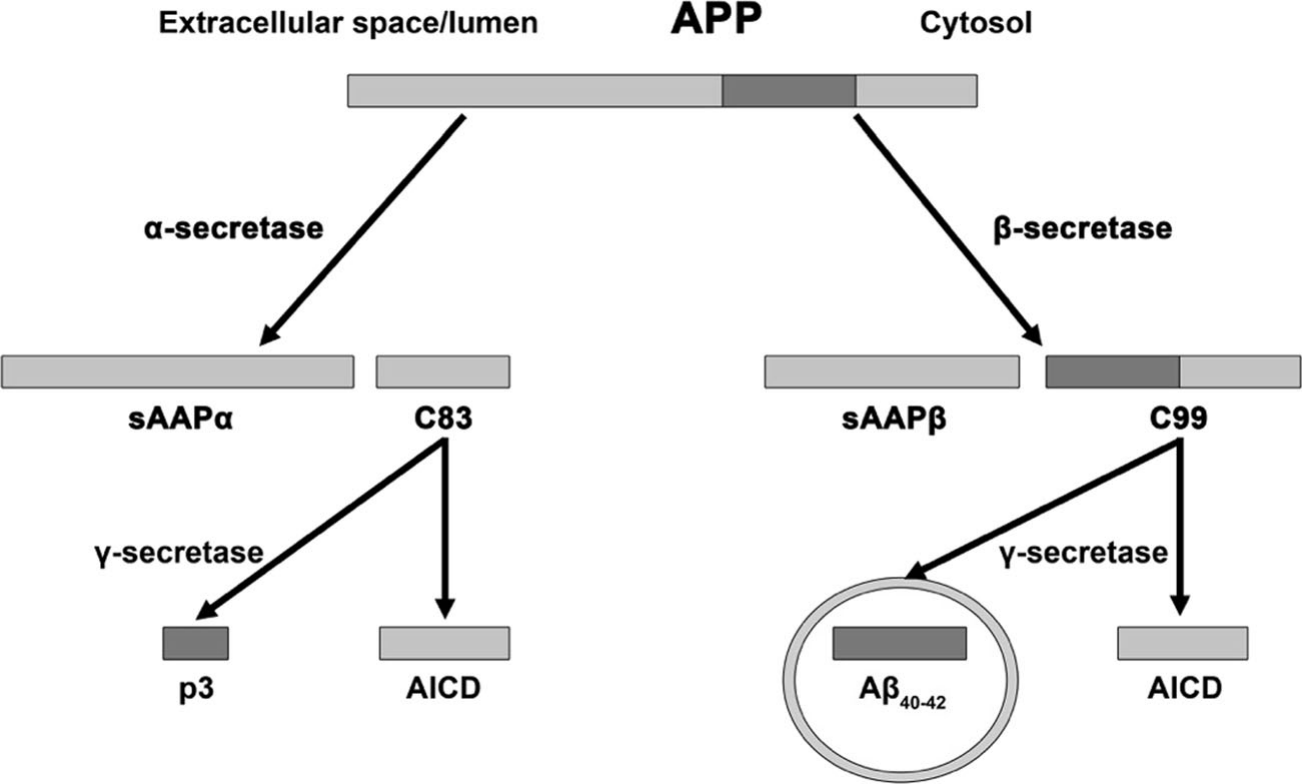
Physiological and pathological APP processing. APP is processed via two mutually exclusive pathways involving cleavage by β-secretase and α-secretase. Cleavage by the latter enzyme intersects the β-amyloid region, which eliminates the possibility of Aβ production and produces membrane-bound C83 protein and sAPPα which enters the cytosol. Subsequent processing of C83 by γ-secretase generates p3 and Aβ together with the amino-terminal APP intracellular domain (AICD). APP cleavage by β-secretase results in the production sAPPβ and C99. Further processing of C99 leads to the production of the AICD fragment and Aβ which forms oligomers and ultimately fibrils

Alzheimer’s disease (AD) is a progressive, clinically heterogeneous, age-sensitive neurodegenerative disease, characterised by often escalating impairments of memory and other cognitive functions together with associated changes in personality and behaviour [1–3]. Amyloid plaques and neurofibrillary tangles (NFTs) are invariant pathological hallmarks seen in the brains of people suffering from of AD [4]. These abnormalities are held to result from the accumulation of small peptides known as amyloid beta (Aβ) in central nervous system (CNS) tissues, and from gross changes in cytoskeletal organisation stemming from the hyperphosphorylation of the microtubule-associated protein tau (ptau) in neurones [5]. According to the classical ‘amyloid cascade’ model of disease causation, Aβ is overproduced following the disruption of homeostatic mechanisms which normally regulate the proteolytic cleavage of the amyloid precursor protein (APP). In this model, age-related genetic and environmental factors conspire to induce a metabolic shift favouring the amyloidogenic processing of APP but inhibiting the physiological, secretory pathway [6–8]. These processes are represented in Figs. 1 and 2 and are well documented and hence will not be the main focus of this paper.

The amyloid hypothesis has been under challenge in recent years as a result of several findings. One is the failure of human trials using therapies targeting the amyloid cascade; another is evidence obtained from positron emission tomography neuroimaging demonstrating increased amyloid accumulation in cognitively intact individuals and an absence of correlation between amyloid load and disease severity in AD patients and in cognitively normal individuals [9, 10].

Hence, while the hypothesis proposing a causative role for Aβ oligomers and ptau as the main, or at least initial, instigator of pathology in AD at least in advanced disease probably holds primacy, there is a growing consensus that the maintenance if not the origin of AD pathology is multifactorial, likely with a high degree of inter-patient heterogeneity [11–14]. This is unsurprising as there is now an extensive body of evidence showing that there are many potential drivers of pathology in the brains of patients diagnosed with AD or mild cognitive impairment (MCI) which are evident in patients with MCI long before the development of amyloid plaques or neurofibrillary tangles (reviewed by [15]). Chronic nitrosative and oxidative stress and significantly depleted levels of reduced glutathione are invariant but non-specific findings, as is the existence of impaired mitochondrial function along many dimensions [16–19].

The presence of activated and dysfunctional microglia and reactive astrogliosis would also seem to be an invariant finding in vivo both in AD and MCI [20–22]. Other commonly reported abnormalities include compromised autophagy and lysosomal clearance accompanied by elevated activity of both glycogen synthase kinase-3 (GSK-3) and mechanistic (previously mammalian) target of rapamycin (mTOR), coupled with a defective ubiquitin-proteasome system (UPS) [12, 23–27]. Several authors have also reported abnormalities in the activity of several kinases and phosphatases, most notably mitogen-activated protein kinases (MAPKs) and protein phosphatase 2A (PP2A or PP2), and transition metal dyshomeostasis, which could all arguably play a role, either as primary or secondary drivers of disease activity [11–13, 16, 28–30].

There is a growing consensus that iron dyshomeostasis plays a pivotal pathological role in the illness, with increased levels of iron proposed as the primary driver of neurodegeneration by many research teams [31–35]. Peripheral immune abnormalities and inflammation are also being increasingly advocated as major, albeit again non-specific, drivers of symptoms [36, 37]. Abnormalities in the composition of the microbiota, and translocation of bacterial antigens into the systemic circulation and the brain, have also become areas of intense research across the neurosciences [38, 39].

Impaired cerebral glucose metabolism is also invariantly reported in AD patients and its occurrence precedes symptoms sometimes for years or even decades [40]. Moreover, the progressive increase in the levels and topography of glucose hypometabolism correlates with an increase in symptom severity and synaptic dysfunction review [40]. In this context, the presence of insulin resistance in AD is unsurprising (reviewed by [41]). This is also concordant with type 2 diabetes mellitus being a risk factor for AD. These observations are of interest as they are common to both disorders and could be explained by the presence of chronic inflammation, oxidative stress and mitochondrial dysfunction in the periphery and brain [42–45]. Chronic inflammation and oxidative stress are also acknowledged causes of GSK-3 and mTOR upregulation and could also account for Aβ upregulation (reviewed by [15]). These observations rather invite the question as to whether increased peripheral inflammation and oxidative stress could be a major driver of the abnormalities repeatedly reported in AD patients. However, it should be noted that these abnormalities have also been repeatedly reported in cognitively intact elderly people as well as in diverse medical and neuropsychiatric disorders [46–54]; hence, there must be other genetic and/or epigenetic factors involved.

Genome-wide association studies (GWASs) have revealed that approximately 40% of AD patients carry the apolipoprotein E (*APOE*) ε4 allele and that *APOE* ε4-positive, but cognitively intact, individuals over 50 years of age are significantly more likely to have brain amyloid deposits than individuals free of that polymorphism [55]; reviewed in [56]. There is also evidence that, compared with age- and sex-matched controls, AD patients carrying both the *APOE* ε4 allele and the H63D polymorphism of the hemochromatosis protein-related class I-like major histocompatibility gene *HFE* are significantly more susceptible to earlier development of AD than those carrying only one of these mutations [57]; reviewed by [58]. More recently, researchers have detected the rs75932628 single-nucleotide polymorphism (SNP) within the triggering receptor expressed on myeloid cells 2 (*TREM2*) gene, leading to an R47H substitution, which increases the risk of developing AD in carriers by virtually the same magnitude as the presence of one *APOE* ε4 allele [59]; reviewed by [60]. However, while genetics clearly plays a role in AD susceptibility, the vast bulk of cases does not show strong genetic underpinnings [61, 62]. Moreover, although common sequence variants in several genes display robust associations with AD susceptibility, evidenced by individual studies and subsequent meta-analyses, collectively, these SNPs only account for approximately a third of attributable risk and the mechanisms underpinning these associations remain undelineated [63]; reviewed by [64].

Recent epigenetic-wide association studies (EWASs) have revealed that AD may be associated with decreased histone acetylation, increased histone phosphorylation (probably including neuronal histone hyperphosphorylation) and DNA hypermethylation with likely increased CpG methylation [62]. Moreover, several research teams have independently reported strong associations between the epigenetic dysregulation of a range of genes and the development of AD in entirely asymptomatic patients (reviewed in [61]). Changes in the methylation status of *ANK-1* which encodes ankyrin repeat domain-containing protein 1, which plays a role in linking integral membrane proteins to the spectrin-actin cytoskeleton, display a particularly strong association with AD development and the burden of neuropathology [65, 66].

Moreover, recent data implicating allele-specific changes to the methylation status of the CpG islands (CGI) responsible for the transcription of *APOE* and downstream genes in AD patients may offer a better understanding of the mechanisms underpinning the increased risk of developing the disease in carriers of the *APOE* ε4 allele [67, 68]. This may also be the case for *TREM2*, as a recent meta-analysis concluded that increased methylation of the *TREM2* promoter region appears to be an invariant feature in the brains of AD patients independently of age and sex [64]. Moreover, this increase in methylation correlates with a higher level of TREM-2 (triggering receptor expressed on myeloid cells 2) activity in the brains of AD patients compared with healthy age- and sex-matched controls [69]. It is also noteworthy that, when viewed as a whole, the results of EWASs indicate that epigenetic abnormalities in tandem with increased levels of inflammation greatly exacerbate the risk of developing AD [61, 65, 66]. In the light of the above, this paper focuses on three questions. First, can genetic and epigenetic factors explain increased levels of peripheral and central inflammation and oxidative stress in AD? Second, could this increased oxidative stress and inflammation originate in the periphery? Third, can the initial development of elevated peripheral and central inflammation and oxidative stress in the context of genetic and epigenetic abnormalities explain the development of AD?

## Evidence of Peripheral Inflammation and Immune Abnormalities in AD

### Evidence of Peripheral Inflammation in AD

Two large meta-analyses have confirmed the presence of elevated pro-inflammatory cytokines (PICs) and other inflammatory molecules in the serum and whole blood of AD patients. In the first of these studies, Swardfager and fellow workers analysed the results of 40 studies and reported a higher inflammatory status, evidenced by elevated levels of interleukin 6 (IL-6), IL-12, tumour necrosis factor-alpha (TNF-α), IL-1β and IL-18, compared with age- and sex-matched healthy controls [37]. These results have been confirmed in a more recent meta-analysis of 175 studies involving 13,344 AD patients and 12,912 healthy controls conducted by Lai and others [70]. These authors reported elevated levels of TNF-α converting enzyme, soluble TNF receptors 1 and 2, IL-6, IL-8, C-X-C motif chemokine-10, IL-2, α1-antichymotrypsin, high-sensitivity C-reactive protein and homocysteine. This meta-analysis also revealed decreased levels of leptin and IL-1 receptor antagonist in AD patients and it is noteworthy that these authors concluded that IL-6 levels were inversely correlated with cognitive scores as ascertained by the Mini-Mental State Examination (MMSE) [70]. The last finding is unsurprising as there is a large body of evidence confirming that inflammatory signals can have a severe adverse effect on brain function, and is consistent with the work of several research teams which have reported that PIC levels in AD patients are positively associated with cognitive decline, increased frequency and severity of neuropsychiatric symptoms, disease severity and overall disease progression [71–76].

It is also worth noting that the combination of PIC levels and brain magnetic resonance imaging (MRI) measures is more predictive of the transition from mild cognitive impairment (MCI) to AD than *APOE* genotype status alone [77, 78]. The weight of data indicates that concentrations of TNF-α in particular appear to have a clear effect on disease progression and/or severity. For example, Holmes and fellow workers reported that a twofold increase in serum TNF-α levels over a 6-month period, indexing successive inflammatory insults, was associated with a twofold rate of cognitive decline over the same period [72]. Furthermore, high baseline levels of the cytokine were associated with a fourfold decline in cognitive function while patients with population-normal levels of TNF-α experienced no cognitive decline over the course of the study [72]. These results were broadly replicated in a later study conducted by the same research team, who reported that TNF-α and IL-6 levels correlated with an increased frequency of neuropsychiatric symptoms characteristic of pathogen-induced sickness behaviour [75]. Finally, a more recent study established a relationship between elevated levels of TNF-α, IL-6 and interferon gamma (IFNγ), produced by abnormally activated T cells, and disease severity [76].

### Evidence of Peripheral Immune Abnormalities in AD

Several research teams have reported abnormalities in CD4 and CD8 T cell activation, differentiation, trafficking and receptor expression in patients with MCI and AD compared with age- and sex-matched controls, although the results reported by different research teams vary [36, 79]; reviewed in [80]. The weight of evidence indicates that CD4 T cells are activated and highly differentiated in AD patients as indicated by a reduction in naïve and central memory CD4^+^ T cells, an increase in Th17 T cells and a reduction in regulatory T cells (Tregs) [34, 81, 82]. In addition, the pattern of receptor distribution on the surface of CD4 T cells may also differ between AD patients and age- and sex-matched controls, with an increased number of CD4^+^ CD28^−^ cells being reported [34]. There is some evidence that the pattern of CD4 T cell activity may be different in patients with MCI compared with AD in whom Treg activity appears to be increased possibly in an attempt to combat increasing inflammation [35].

The data regarding various aspects of CD8 T cell abnormalities in AD patients are mixed and often conflicting with increased numbers and activity, decreased numbers and activity and no changes compared with age- and sex-matched controls all being reported [34, 36, 83]. However, several authors have suggested that these inconsistencies could potentially be explained by the different methods used and differences in compartments sampled [80].

The pathogenic significance, if any, of these T cell abnormalities is still a matter of debate but there is a growing body of evidence that the entry of activated CD4 and CD8 T cells into the CNS and dysfunctional ‘cross talk’ between the CNS and the peripheral immune system make a significant contribution to the genesis and/or exacerbation of pathology in at least some patients with AD [84, 85]. In this context, it is noteworthy that several research teams have reported the presence of CD4 and CD8 T cells in the brains of AD patients post mortem (reviewed by [86]) and a recent study has reported a significant correlation between the extent of CD8 T cell activation and parahippocampal microstructural tissue damage in AD patients [83]. Moreover, this last team of authors reported that levels of activated HLA-DR-positive CD4^+^ and CD8^+^ T cells were significantly increased in the peripheral blood of AD and MCI patients compared with age- and sex-matched controls, but not in patients with a range of non-AD dementias [83]. This finding is consistent with that of other published research which indicates that the pattern of T cell abnormalities seen in AD may well be specific to the disease [87, 88].

### Potential Origins of Peripheral Inflammation and Immune Activation in AD

#### The Presence of Serum Aβ Autoantibodies

The origin of the chronic peripheral activation and activated but dysregulated immune system seen in AD patients has not been delineated, but the presence of autoantibodies directed at Aβ in the serum of AD patients, possibly as a result of efflux from the brain, should be considered as certain classes of antibody are well-documented inflammatory mediators [36]. The evidence regarding the existence of increased levels of these antibodies in AD patients compared with age- and sex-matched controls is unconvincing, however, with elevated levels, reduced levels and no significant differences being reported (reviewed by [89]). It is also worthy of note that the levels of B cells producing autoantibodies against Aβ appear to be the same in AD patients and healthy controls [90, 91]. Moreover, thus far, all available evidence demonstrates that these autoantibodies (both IgM and IgG) are catalytic in nature, meaning that they rarely form stable complexes and are not recognised sources of inflammation [92, 93]. The lack of association between serum Aβ autoantibody levels and Aβ levels in the brain reported by Xu and others is also relevant as this finding casts doubt on the origin of serum Aβ [91]. The lack of T cell responses to Aβ in AD patients reported by Baril and colleagues is also pertinent; this finding renders the hypothesis that antibodies to Aβ in the serum of AD patients are the cause of T cell activation and differentiation patterns in such patients improbable, although it cannot be ruled out [94].

#### Dysbiosis and Translocation of Commensal Lipopolysaccharide

Another possible cause could stem from disturbances in the composition of the microbiota and translocation of commensal LPS into the peripheral circulation, which have both been recently reported in AD, although this again is a very non-specific finding [38, 39]. The inflammatory consequences of this latter phenomenon, achieved via activation of toll-like receptors on the surfaces of macrophages and dendritic cells and the subsequent production of PICs, are well documented and hence bacterial translocation as a consequence of increased intestinal permeability could go some way to explaining chronic systemic inflammation in AD (reviewed by [95, 96]).

Increased levels of translocated LPS can also have profound effects on T cell activation, differentiation and trafficking, and thus could potentially explain at least some of the peripheral T cell abnormalities seen in AD patients. For example, LPS activation of antigen-presentation cells (APCs) via TRIF (TIR (toll/IL-1 receptor) domain-containing adaptor-inducing IFNβ)- and MyD88 (myeloid differentiation primary response 88)-dependent signalling pathways initiates CD4 T helper cell clonal expansion and differentiation [97]. The effect of LPS exposure on CD4 T cell differentiation appears to be tissue dependent as evidenced by reports of Th1 cell differentiation being induced by the presence of LPS in lymphoid tissue and Th17 cell differentiation being the result of naïve CD4 T cell exposure to LPS in the intestinal lamina propria [97]. LPS also affects T cell differentiation indirectly by stimulating B cells via a mechanism involving toll-like receptor-4 (TLR-4) and B cell-activating factor belonging to TNF superfamily (BAFF) activation, which results in naïve CD4 T cell differentiation towards a Th2 or Treg lineage depending on localised levels of that commensal antigen [98]; reviewed in [99]. Finally, it has been suggested that activation of TLR-4 receptors on CD4 T cells by LPS may predispose to the development of autoimmunity as such activation appears to increase the proliferation and inflammatory status and survival of Th1 and Th17 cells [100].

Translocated LPS would also appear to exert a range of effects on CD8^+^ T cell activation, differentiation, survival and trafficking. For example, Cui and fellow workers reported increased proliferation and survival of memory CD8^+^ T lymphocytes in an environment of high LPS, while McAleer and others reported increased CD8^+^ T cell trafficking into non-lymphoid tissue under similar conditions [101, 102]. The surface TLR-4 receptors are directly sensitive to the presence of LPS and thus evidence demonstrating their activation in an environment of high LPS, as characterised by elevated levels of CD25 and CD69 receptors, in the absence of APC activation, is unsurprising [103]. This interaction would appear to be of considerable pathogenic importance in vivo and is now considered to be a major driver of tissue damage in rheumatoid arthritis [104], which is of interest given the data implicating increased CD8^+^ T cell activation levels and numbers as a driver of tissue damage in AD as described above.

There is evidence to suggest that LPS also induces synthesis of IFNγ by natural killer (NK) cells via a mechanism which does not appear to involve TLR-4 activation on APCs [105], and there are replicated data indicating that the presence of this antigen stimulates the proliferation of CD56^+^ CD3^−^ NK cells, which appear to play a role in the pathogenesis of AD [106, 107]; reviewed in [108].

APOE plays a regulatory role in inflammatory signalling in APCs and there is some evidence to suggest that the *APOE* ε4 allele is associated with higher levels of PIC production by LPS-activated macrophages via upregulation of NF-κB transcription resulting in increased levels of TNF-α and IL-1 with a concomitant reduction in IL-10, which is of interest given the probable role of translocated LPS in the aetiology of peripheral inflammation in AD discussed above [109, 110].

Cash and colleagues studied mice in which the endogenous *apoe* gene was replaced, at the same locus, by either the human *APOE4* or *APOE3* gene; compared with the *APOE3* mice, the *APOE4* ones showed defective macrophagic efferocytosis, which is a process involving the phagocytosis and immunologically silent clearance of dying and dead cells [111]. This may have significant pathological consequences given considerable data indicating that tissue inflammation may result from the failure of this mechanism; impaired efferocytosis is being increasingly implicated in the pathogenesis of autoinflammatory and autoimmune diseases [112].

The presence of dysbiosis in AD patients, which seems to involve increased Bacteroidetes, decreased Firmicutes and Actinobacteria (including decreased *Bifidobacterium* and *Adlercreutzia* genera) phyla compared with age- and sex-matched controls [38], may also contribute to the Th17/Treg imbalance reported in AD, as described above. Several research teams have independently reported that the composition of the microbiota plays a key role in determining the trajectory of activated naïve CD4 T cell differentiation along the Th17 or Treg pathways [113, 114]. It should be noted that there are few Th17 cells in lymph nodes and the vast bulk of this T cell population resides in the intestinal lamina propria and can home in to the blood and other peripheral tissues following activation, and therefore can be a source of the systemically elevated T cells of this type reported in AD [95, 115]. Intriguingly, there is also accumulating evidence suggesting that gut microbiota profiles influence the DNA methylation patterns of T cells and other cellular inhabitants in the blood, thereby determining, at least to some extent, the inflammatory status of an individual [116].

This is a complex area and readers interested in pursuing this matter are invited to consult an excellent and comprehensive review conducted by Ye and fellow workers [117]. The class of apolipoprotein E (ApoE) proteins plays a major role in regulating intestinal immune system homeostasis, colonic inflammation and composition of the microbiota, and therefore it is tempting to speculate that *APOE* ε4 allele status could be associated with pathological changes in all these parameters; however, it must be stressed that there is no evidence regarding this area in AD or indeed any other disease [118].

#### Epigenetic Changes in Peripheral Mononuclear Blood Cells

There is evidence of epigenetic dysregulation in the T cells and macrophages of AD patients compared with age- and sex-matched controls, with increased expression of microRNA-155 (miR-155) being reported in T cells and differential DNA methylation changes being observed in CD14^+^ macrophages [119, 120]. These findings could also potentially indicate a source of elevated peripheral inflammation in AD as miR-155 is NF-κB sensitive and also acts to increase the transcription of NF-κB, which in turn allows for increasing levels of inflammation and PIC production by activated T cells in a positive feedback loop [121, 122]. While the origin of increased expression of miR-155 in AD is not known, one potential cause could be translocated LPS which is documented to increase the expression of this molecule in human peripheral mononuclear blood cells (PMBCs) most notably macrophages [123].

There is also an accumulating body of evidence implicating epigenetic dysregulation, most notably increased DNA methylation and altered miRNA expression, with an elevated inflammatory status of macrophages [124, 125]. For example, Wang and colleagues reported that obesity-induced hypermethylation of DNA in the promoter region of peroxisome proliferator-activated receptor γ1 (PPARγ1) exacerbated the inflammatory status of macrophages and provoked a polarisation towards the M1 phenotype [124]. These findings were also reported by Yang and fellow workers in an earlier study [126]. miRNA profiles and levels also regulate the inflammatory status and polarisation of macrophages via several different mechanisms including NF-κB transcription and cellular location [127, 128].

*APOE* allele status is a major influence on miRNA expression patterns in macrophages [125]. These authors reported that 152 miRNAs were differentially expressed in murine macrophages over-expressing ApoE4 compared with those over-expressing ApoE3. The differential elevation of mir-146a and miR-21 may be significant as they are associated with increased matrix metalloproteinase-9 (MMP-9) production and a corresponding increase in macrophage-associated tissue damage [125]. The upregulation of miR-146a may be of particular pathological relevance as this molecule may be upregulated by IL-1β, TNF-α or LPS, and increased activity of this miRNA is associated with increases in the activity of numerous inflammatory pathways in AD (reviewed in [129]).

#### Influence of TREM-2 Elevation in PMBCs

TREM-2, although better known as a regulator of microglial function as will be discussed below, also regulates TLR responses on dendritic cells. *TREM2* upregulation in such cells appears to accelerate their maturation and trafficking to lymph nodes and sites of infection, as well as stimulating the differentiation of Th2 or Th17 cells, dependent on the nature and concentration of the antigen presented [130, 131]. The fact that TREM-2 acts as an ApoE receptor may also be of importance as this allows for an exaggerated effect of the ApoE4 protein in the context of dysfunctional TREM-2 receptors [132]. Moreover, a recent meta-analysis reported that increased methylation of the *TREM2* promoter region appears to be an invariant feature in the brains of AD patients independently of age and sex [64]. Moreover, this increase in methylation is associated with a higher level of TREM-2 activity in the brains of AD patients compared with healthy controls [69]. Increased expression of TREM-2 receptors on peripheral leucocytes of AD and MCI patients, associated with reduced methylation in *TREM2* intron 1, has been consistently reported [133–135]. Tan and colleagues have investigated the relationship between increased expression of *TREM2* mRNA in the periphery in AD patients, and their study appears worthy of particular consideration as their results appear to emphasise the importance of peripheral abnormalities in the development of neuropathology in AD and partly to explain the relationship [135]. Briefly, these authors reported highly significant negative correlations (controlling for age, sex, ethnicity and *APOE* allele status) between, on the one hand, *TREM2* mRNA expression (following amplification by real-time quantitative polymerase chain reaction (qPCR)), and, on the other hand, MMSE score residuals, episodic memory score residuals and Montreal Cognitive Assessment (MoCA) score residuals; there was also a negative correlation with right hippocampal volume and with the grey matter (GM) volumes of the frontal, temporal and parietal cortices [135]. Their analyses also revealed that, following a median split according to MoCA scores, compared with controls those AD patients in the lower group (MoCA scores ≤ 20) had higher *TREM2* mRNA expression, which correlated with reduced volumes of total GM and right and left hippocampi [135].

#### Effect of PP2A Inhibition

Finally, it is also noteworthy that PP2A inhibition, which also appears to be a universal feature in AD patients [29], may lead to exacerbated PIC production by LPS-activated APCs [136, 137]. The mechanism underpinning this phenomenon has not been fully delineated but it appears to be associated with altered levels of histone post-translational methylation leading to increased transcription of *TNFA* (the TNF-α gene) and a general increase in inflammatory status [137]. These findings, allied to those discussed above, may well be important from the perspective of AD pathogenesis as the association between peripherally increased PICs and TREM-2 and increased AD risk and/or severity could be explained by the initiation and/or exacerbation of microglial activation, either as a result of high peripheral PIC levels and/or the egress of activated Th1 and/or Th17 cells into the CNS. Readers interested in the details of the mechanisms involved are referred to these reviews by Morris and colleagues [138] [139]. The pathological consequences of microglial activation and dysfunction and the putative role of these glial cells in the pathogenesis and pathophysiology of AD are discussed below.

## Role of Microglia and Astrocytes

It should be stated at the outset that much of the data regarding the role of microglia in AD has been obtained from in vitro and non-human animal studies or AD patients post mortem, and their role is still a source of debate [140]. However, the use of in vivo neuroimaging techniques has consistently revealed a pattern of microglial activation consistent with an increased inflammatory status. For example, Parbo and colleagues reported the presence of increased cortical microglial activation in 85% of their MCI cohort [141]. Moreover, these authors noted that the patterns and extent of microglial activation correlated with the patterns and level of amyloid load in the parietal, frontal and temporal cortices [141]. Fan and co-workers also reported significantly elevated microglial activation at baseline in their AD participants, which increased in the majority of the patients over the course of the study [21]. Moreover, these authors reported that this longitudinal increase in microglial activation correlated with amyloid deposition and decline in regional cerebral metabolic rate over time [21]. In a later study, this team of authors investigated longitudinal changes in microglial activity in MCI and AD patients and reported a 36% increase in microglial activation over 14 months in the AD patients but an 18% decrease in the MCI patients for reasons which are not currently understood [20].

These findings are consistent with those of the work of other authors who have produced evidence suggesting that such microglial activation and subsequent production of cytokines, chemokines, nitric oxide (NO), prostaglandins, reactive oxygen species (ROS), inducible nitric oxide synthase (iNOS) and cyclooxygenase-2 (COX-2), and other mediators of inflammation and neurotoxicity also play a critical role in AD pathogenesis [142–144]. The weight of evidence suggests that microglia enter a hyper-reactive state in AD, and indeed other neurodegenerative conditions, and lose their normal beneficial function in maintaining neuronal homeostasis and phagocytic clearance during the progression of the illness [145], and ultimately adopt a neurotoxic or ‘primed’ phenotype [71, 146]. It has been proposed that this primed or hyper-responsive phenotype, which leads to an exaggerated production of neurotoxic substances following inflammatory activation, is the result of successive immune or inflammatory insults in the periphery [22, 147]. The activation of microglia, and the ultimate creation of a hyper-responsive phenotype, would also go some way to explaining the wealth of experimental data demonstrating that systemic inflammation, such as that resulting from pathogen invasion, can worsen the symptoms of AD or even trigger its development [142]. Unsurprisingly, there has been intensive research investigating the mechanisms underpinning microglial pathology in AD and currently, a great deal of research is focused on TREM-2, which is considered below.

### Abnormalities in TREM-2 Levels and Function as a Source of Microglial Pathology

As mentioned above, a recent meta-analysis concluded that increased methylation of the *TREM2* promoter region appears to be an invariant feature in the brains of AD patients independently of age and sex [64]; furthermore, this increase in methylation is associated with a higher level of TREM-2 activity in the brains of AD patients compared with healthy age- and sex-matched controls [69]. Moreover, there is a wealth of data demonstrating that functional variants of the *TREM2* gene are strongly associated with an increased risk of late onset AD (LOAD) development [148, 149].

Increased TREM-2 activity in AD brains may be a significant source of pathology as this receptor plays a major role in regulating microglial activation and the inflammatory response following TLR activation, and facilitates immunologically silent phagocytosis of apoptotic neurones [150, 151]. Increased *TREM2* expression in the temporal cortex of AD patients post mortem correlates significantly with increases in caspase-3 and phosphorylated-tau, and intense TREM-2 immunoreactivity is seen in microglia associated with amyloid plaques in regions of profound neuritic pathology [152]. This and other data have led to the proposal that *TREM2* variants contribute to the development of Alzheimer’s disease via the downregulation of microglial Aβ phagocytic capability and dysregulation of microglial pro-inflammatory responses [151]. The relationship between TREM-2 and the development of a neuroinflammatory state appears to be complex and appears to involve improving microglial survival and metabolic performance as well as stimulating the release of PICs, ROS, reactive nitrogen species (RNS) and PGEs [60]. There is also some evidence to suggest that increasing levels of neuroinflammation provoke further upregulation of the TREM-2 receptor on activated microglia allowing for an upwards spiral of inflammation via a positive feedback loop [153]. In addition, TREM-2 acts as an ApoE receptor [132], as discussed above, and in this context, it is noteworthy that recent studies have established a relationship between TREM-2 and ApoE in the regulation of the microglial phenotype and the level of inflammatory mediators excreted by these glial cells following activation [154, 155]. In particular, evidence suggests that ApoE-mediated TREM-2 signalling provokes a change in microglial phenotype from tolerogenic to neurodegenerative following phagocytosis of apoptosed neurones in vivo [154] and the presence of the ApoE4 isoform is associated with higher levels of neuroinflammation in such circumstances by differentially increasing levels of TREM-2 [155].

TREM-2 activity is also intimately connected with microglial phagocytosis as discussed above and exerts its signalling effects via a multi-receptor complex with signalling adaptor molecule DNAX-activating protein of 12 kDa (DAP12) and dysfunction of this signalling axis may play a role in the impaired microglial phagocytosis repeatedly reported in AD brains. Briefly, under physiological conditions, heat shock protein-90 (HSP-90) engagement with TREM-2 regulates the immunologically silent microglial phagocytosis of apoptotic neurones via engagement with DAP12 [152]. The protective effect of TREM-2 against the development of LPS-mediated neuroinflammation would also appear to be mediated by this route [156]. Given such information, the existence of data suggesting that functional mutations in either protein can have adverse effects on microglial phenotype and function is unsurprising and may be one factor accounting for the impaired microglial phagocytosis which appears to be a feature of AD [157, 158].

The physical association between TREM-2 and DAP12 plays a vital role in determining the outcome of TREM-2 activation and in particular anti-inflammatory consequences are dependent on DAP12-mediated stabilisation of the C-terminal fragment of TREM-2 (CTF) and the loss of physical contact has pro-inflammatory consequences [156]. This is of importance as there is evidence to suggest that CTF accumulation in AD leads to disconnection between TREM-2 and DAP12, which could provide a mechanism to explain impaired phagocytosis and the pro-inflammatory consequences of TREM-2/DAP12 signalling in AD and a range of other neurodegenerative diseases [158–160].

### Epigenetic Dysregulation of ANK1 as a Source of Microglial Pathology

The pivotal role of microglial pathology in the pathogenesis of AD has been further highlighted by research into the methylation status of the gene *ANK1*, the expression of which in AD brains in vivo appears to be confined to these glial cells [61]. Briefly, two independent research teams have reported the presence of a hypermethylated region in *ANK1* and changes in *ANK1* mRNA levels are associated with the geographical extent and overall burden of neuropathology in the entorhinal cortex, prefrontal cortex and superior temporal gyrus in symptomatic and pre-symptomatic AD patients in post mortem studies [65, 66]. These are important observations: they are relatively large studies and the methylation changes seen in asymptomatic patients are unlikely to be the product of disease pathology [161]. It should also be noted that the association between AD pathology and *ANK1* methylation status may well be the most robust of all epigenetic and genetic associations with disease development reported thus far [61, 161].

The mechanisms underpinning this association are not understood, but they may be connected to altered expression of miR-486. *ANK1* is a host gene for miR-486 [162] which in turn is a source of two mature miR-486 miRNAs, namely, miR-486-3p and miR-486-5p [163]. Importantly, *ANK1* hypermethylation inhibits the transcription of miR-486 [164], which may have pathogenic consequences as suppression of this miRNA has pro-inflammatory consequences and is furthermore associated with increased cellular survival and proliferation [165, 166]. Furthermore, upregulation of miR-486 acts as a negative regulator of Akt (protein kinase B), mTOR and STAT3 (signal transducer and activator of transcription 3), all of which play major roles in microglial activation, proliferation and survival [167, 168].

mTOR plays a pivotal role in determining the inflammatory status and proliferation of microglia following PIC-mediated activation, which are both key determinants of neuroinflammation [169]. Akt upregulation is also a major driver of microglial activation and polarisation into the M1 phenotype [170]. STAT3 activation also plays a major role in determining the magnitude of the proliferative and inflammatory responses of activated microglia and, crucially, activation of this transcription factor also inhibits the microglial phagocytosis and clearance of Aβ in vivo [171, 172]; reviewed by [173]. Thus, it is conceivable that *ANK1* hypermethylation accounts for the elevated mTOR and STAT3 signalling which has been repeatedly documented in the microglia of AD patients [172, 174–176].

### Role of Aβ in Microglial Pathology

This is a well-documented area and has been considered by numerous authors (e.g. [15, 177]). It seems reasonable to propose that accumulating levels of Aβ as a result of impaired clearance would also play a role in maintaining microglia in a chronic state of activation following antigenic stimulation via engagement with TLR-2, which mediates antigenic stimulation of these glial cells by this peptide [178]. However, the capacity of Aβ to activate microglia in vivo has not been demonstrated and several authors have noted that human brains with very high Aβ loads reveal an absence of microglial activation [140, 179].

### Interactions Between Microglia and Astrocytes and Exacerbated Neuroinflammation

Early AD is characterised by astroglial atrophy leading to impaired blood-brain barrier (BBB) structure and function, synaptic dysfunction, mitochondrial dysfunction and impaired neuronal homeostasis [180–182]. Later disease is associated with reactive astrogliosis where activated astrocytes make an independent contribution to increasing neuroinflammation and neurotoxicity [180, 181]. Astrocytes make many contributions towards brain homeostasis in the context of AD, including regulation of oxygen and energy delivery to neurones, regulation of cholesterol delivery to neurones, neurotransmission, and immune and inflammatory responses in the CNS, in a similar manner to its activity in the periphery discussed above [182]. This is highly relevant because astrocytes are by far and away the largest producers of ApoE in the brain and ApoE4 is known to impair BBB function to a greater extent than other ApoE isoforms [183, 184]. Activated astrocytes are also a source of Aβ_42_ protofibrils, likely synthesised by the actions of PICs [185, 186]. There is also some evidence that reactive astrocytes in AD not only secrete increased levels of Aβ_42_ as discussed above but also conspire with adjacent neurones to promote further increases in Aβ_42_ and levels of ptau over a wider geographical area as the disease progresses [187].

There are a number of mechanisms which could account for the greater levels of peripheral inflammation and neuroinflammation that occurs in AD patients than in age- and sex-matched controls, which in turn appear to make a significant contribution to the development of AD, and it is certainly plausible that the development of AD begins with pathology in the periphery. It should also be noted that, while the data reviewed above focus heavily on inflammation, this phenomenon is invariably accompanied by oxidative stress [188, 189]. Hence, the mechanisms potentially explaining differentially elevated inflammation in the brain and periphery of AD patients also potentially explain elevated oxidative stress in both compartments. This is an important point as the remainder of the paper focuses on the third research question, namely, whether differentially elevated inflammation and oxidative stress in the brain and periphery of AD patients is sufficient to explain impaired mitochondrial function, synaptic dysfunction, PPA2 inhibition, elevated mTOR, elevated GSK-3, impaired macro- and microautophagy, decreased proteasome function, increased iron accumulation and transition metal dyshomeostasis reported in AD patients compared with age- and sex-matched controls.

## Evidence and Consequences of Chronic Oxidative Stress in AD

### Evidence of Increased Oxidative Stress in the Brain and Periphery in AD Patients

Surrogate markers of protein oxidation, lipid peroxidation and oxidative damage to DNA, such as protein carbonyls, 3-nitrotyrosine, malondialdehyde (MDA), 4-hydroxynonenal, F_2_-isoprostanes, 8-hydroxydeoxyguanosine (8-OHdG) and 8-hydroxyguanosine, are elevated in the cerebrospinal fluid, the brain and peripheral cells of patients with AD [16, 190, 191]. Damaged proteins, lipids, RNA and DNA in regions of the brain associated with cognitive function are also a reproducible finding in AD and MCI and are held to have a functional role in disease pathogenesis [192, 193].

### Effect of Oxidative Stress on the Development of Amyloid and Tau Pathology

Oxidative stress not only impairs the activity of α-secretase but also enhances the activation and expression of β- and γ-secretase [194, 195]. The oxidative stress-driven stimulation of β-secretase 1 (BACE1) and presenilin-1 (PS1) activities, and the activation of γ-secretase, are dependent on the NF-κB and activator protein 1 (AP-1)-induced activation of the c-Jun N-terminal kinase (JNK) pathway [196, 197]. In essence, the promoter region of the *BACE1* gene hosts binding sites for the redox-sensitive AP-1 and NF-κB; the activation of which in an environment of chronic oxidative stress explains the enhanced transcription of *BACE1* [198], elevated JNK signalling [16], increased expression of *BACE1* and increased PS1 activity, which have been detected in AD brains [199–201]. Hence, NF-κB- and AP-1-induced activation of JNK signalling, and consequent upregulation of *BACE1* and *PSEN1* (which encodes PS1), likely could lead to increased Aβ production and possibly an exacerbation of cognitive decline and neuronal apoptosis in AD [16, 200].

There is also an accumulating body of evidence indicating that chronic oxidative stress has a direct causal role in tau phosphorylation [202–204]. The mechanisms underpinning these observations remain to be fully elucidated but the weight of evidence implicates elevated levels of fatty acids and p38 signalling [204–206].

### Other Pathological Consequences of Elevated Oxidative Stress

Signs of oxidative and nitrosative damage to proteins and lipids are amongst the earliest indicators of early disease and occur before evidence of Aβ accumulation [28]. A study comparing F_2_-isoprostane levels in the frontal poles of AD brains with the same regions from brains of patients with schizophrenia and Parkinson’s disease (PD) reported no differences between PD, schizophrenia and controls, but the levels were significantly increased in AD which potentially allows for higher levels of oxidative stress in those brain areas as a unique contribution to the pathogenesis of the illness [207].

Oxidative stress has also been associated with *APOE* status in AD patients and interestingly also in healthy subjects [28]. In particular, the *APOE* ε4-positive status is associated with a relatively higher level of oxidative stress and diminished antioxidant enzyme activity in the hippocampus of AD patients [208]. The association with *APOE* status is not surprising as ApoE is a key player in organising cellular antioxidant responses [209]. The levels of oxidative stress in peripheral lymphocytes are also higher in AD patients with at least one copy of the *APOE* ε4 allele [210]. It is also of interest that *APOE* ε4 directly facilitates the phosphorylation of tau, potentially increasing the filamentous load of this protein in the brain in AD [211].

### Oxidative Stress and the Development of Mitochondrial Dysfunction in AD Patients

Extensive studies have demonstrated that mitochondrial dysfunction is an important factor involved in the pathogenesis of AD and is apparent in the earliest stages of the disease both in the brain and the periphery [19, 212]. Several studies have identified structural and functional mitochondrial abnormalities in hippocampal neurones of AD patients compared with age- and sex-matched controls [213–216]. Such abnormalities include a significant reduction in mitochondrial numbers and exaggerated levels of oxidised mitochondrial DNA (mtDNA) and nitrated proteins in the cytoplasm in a pattern suggestive of impaired mitophagy or fission dynamics [215–217]. These mitochondrial abnormalities are accompanied by oxidative damage marked by 8-hydroxyguanosine and nitrotyrosine, indicating that the mitochondria are damaged by ROS and RNS during disease progression [213, 218, 219].

Several authors have reported decreased mitochondrial complex IV activity in the frontal cortex of AD patients and this phenomenon leads to increased ROS production and depleted adenosine triphosphate (ATP) production, contributing to neuronal dysfunction and, ultimately, degeneration [213, 214, 217]. Systemic mitochondrial dysfunction is also apparent in all phases of the illness, as evidenced by impaired mitochondrial electron transport chain (ETC) activity and depleted basal mitochondrial membrane potential, seen in PMBCs of patients with AD and MCI [220–222]. In this context, it is noteworthy that high levels of NO have a well-documented inhibitory effect on ETC enzymes as a result of *S*-nitrosylation of key functional cysteine residues in their catalytic sites (reviewed in [223]). Oxidative and nitrosative stress can also lead to oxidative damage to key enzymes of the tricarboxylic acid (Kreb’s) cycle, leading to their inactivation, which exerts a range of unfavourable effects on cellular bioenergetics. These are well documented phenomena and will not be considered further here. The interested reader is referred to the works of Morris, Maes and Praticò [207, 224] for details of these mechanisms. It is, however, worthy of note that mitochondrial dysfunction leads to dramatically elevated levels of ROS and NO production, which further compromise mitochondrial function, leading to a vicious spiral of mitochondrial damage and bioenergetics failure [225, 226].

There is accumulating evidence that *APOE* status has an effect on mitochondrial function in at least some patients with AD. Gibson and colleagues reported that mitochondrial dysfunction was more common in the brains of AD patients with the *APOE* ε4 allele [227]. The mechanisms explaining this association are not completely understood but there is some evidence to suggest that the neurotoxicity stems at least in part from the entry of ApoE4 isoform fragments into the cytosol and ultimately into mitochondrial membranes [184]. Once in situ, this lipoprotein induces mitochondrial dysfunction by binding to the α- and β-subunits of the mitochondrial F1-ATPase and disrupting mitochondrial membrane integrity leading to dissipation of the trans-membrane potential difference [184, 191]. There is also some evidence that the *APOE* ε4 allele and mtDNA haplogroups are cooperative variables in the sporadic form of AD [228].

In addition, accumulating data indicate that changes in methylation status of the promoter region of the *APOE* gene in AD patients can have a direct influence on mitochondrial function and indeed the development of mitochondrial pathology [67, 68]. In brief, the methylation status of a CGI in the 3′ region, 2.6 kb downstream of the *APOE* promoter, modulates *APOE* expression. Moreover, common *APOE* SNPs reside in this region and can regulate levels of methylation and transcription in an allele-specific manner with ε4 having a greater effect than ε3. These methylation changes not only influence the transcription of *APOE* but also affect that of *TOMM40* encoding the mitochondrial protein translocase of outer mitochondrial membrane 40 homolog (TOMM40), which plays an essential role in the importation of proteins into the organelle [67, 68]. This is significant given that a recent study has reported reduced levels of TOMM40 in the brains of AD patients which correlated with the extent of cognitive decline [229] and the results of a large meta-analysis involving 10,358 AD cases and 18,157 healthy controls which concluded that the *TOMM40 rs2075650* polymorphism was associated with an increased risk of disease development (odds ratio 4.178) [230]. The mechanisms underpinning reduced TOMM40 expression and/or conformational changes to this protein and the development of AD and other neurodegenerative diseases are discussed by Gottschalk and colleagues [231]. Lastly, there is evidence that mitochondrial dysfunction might be worsened by neuronal accumulation of oligomeric Aβ (OAβ).

## Oxidative Stress and the Development of Synaptic Dysfunction

Numerous research teams have adduced evidence supporting a direct causal relationship between oxidative stress and the development of synaptic dysfunction in AD [232]; reviewed by [233]. This is also true of mitochondrial dysfunction and glucose hypometabolism which is apparent in the posterior cingulate cortex and other AD-vulnerable brain regions in MCI patients and healthy adult carriers of *APOE* ε4 many years or even decades before the development of clinical symptoms and, crucially, before any discernible evidence of tau or Aβ pathology [234, 235]; reviewed by [236]. The origin of glucose hypometabolism, which appears to be an invariant feature in the brains of AD patients [40], is a subject of debate with some authors suggesting that this phenomenon is secondary to mitochondrial dysfunction [44] while others cite as the cause of brain insulin resistance, which is also an invariant feature in AD patients [41]. It is also of interest that the insulin resistance seen in AD patients could be the result of chronic oxidative stress [45, 237], indirectly associating chronic oxidative stress with the development of glucose hypometabolism [238, 239].

The association between impaired mitochondrial performance and the development of synaptic dysfunction is not unexpected as these organelles are involved in every stage of neurotransmission including the synthesis and storage of neurotransmitters, the trafficking and recycling of synaptic vesicles (SVs), presynaptic neurotransmitter release, neurotransmitter synthesis, calcium ion homeostasis as well as supplying ATP and regulating levels of ROS [240–242].

Mechanisms underpinning the detrimental effects of excessive ROS levels on synaptic function are underpinned by oxidation of cytosolic and membrane proteins and peroxidation of membrane lipids [243, 244]. For example, several research teams have reported that lipid peroxidation in presynaptic membranes impedes fusion pore opening, thereby restricting SV exocytosis, resulting in the abnormal retention of SVs within presynaptic active zones [245, 246]. The last phenomenon may go some way to explaining the presence of data demonstrating attenuation of synaptic neurotransmission and long-term potentiation (LTP) by high levels of ROS (reviewed by [247]).

More specifically, increasing levels of ROS and RNS could account for the progressive loss of cholinergic neurones and increasing dysfunction of cholinergic neurotransmission which are characteristic of AD patients as their disease progresses [248]. For example, the enzyme choline acetyltransferase (ChAT) and the high-affinity choline transporter (CHT), the enzymes responsible for synthesising and recycling acetylcholine (ACh), respectively, are vulnerable to post-translational modifications leading to compromised trafficking and protein-protein interactions or indeed inactivation by the effects of ROS and ROS owing to a high density of essential cysteine residues in enzyme catalytic sites (reviewed by [249]). Moreover, acetylcholinesterase (AChE), an enzyme responsible for ACh hydrolysis in the synaptic cleft, is also prone to inhibition in such an environment [250, 251]. Furthermore, there is a considerable body of evidence indicating that cholinergic neurones are highly susceptible to apoptotic or necrotic death in an environment of excessive nitrosative and oxidative stress [252, 253] via mechanisms detailed in a recent paper by Morris and fellow workers [139]. Readers interested in a detailed consideration of cholinergic neurotransmission and the role of the molecular players described above in the context of AD are invited to consult an excellent review by Ferreira-Vieira and fellow workers [248].

The existence of synaptic dysfunction in AD may also be influenced by inhibition of glutamatergic *N*-methyl-d-aspartate (NMDA) receptors, which has been repeatedly reported in AD patients [254], via oxidation of cysteine groups on key structural and functional proteins leading to profound changes in conformation and function [223, 255]. NMDA receptor function can also be compromised by high levels of NO through hypernitrosylation of key receptor subunits [226] and via the formation of the excessively damaging peroxynitrite [256].

While the association between increased oxidative stress and increasing bioenergetic dysfunction, as evidenced by increasing glucose hypometabolism, increased lactate and pyruvate and the development of increasing synaptic dysfunction seen in preclinical AD and *APOE* ε4 carriers, is not associated with Aβ accumulation [257] (reviewed by [258]), recent research suggests that this might not be the case for tau deposition although findings are mixed [259–261]. For example, Bischof and others and Kang et al. reported a positive correlation between tau deposition and glucose hypometabolism in cross-sectional studies [259, 261]. However, Chiotis and colleagues concluded that increases in glucose hypometabolism were not associated with increased tau deposition in a large longitudinal study [260].

## Increased Oxidative Stress and Altered GSK-3 Activity in AD Patients

Prolonged and severe oxidative stress leads to the activation of GSK-3 [262–264]; its physiological levels of expression and activity play an indispensable role in the regulation of synaptic function and other aspects of neurotransmission as well as levels of tau phosphorylation [12, 265]. Given this information, the presence of data implicating dysregulation in the activity of the two isoforms of this kinase as one cause of synaptic dysfunction in MCI and AD is unsurprising (reviewed by [266]).

The weight of direct and indirect evidence suggests that GSK-3 production is increased in the hippocampus and frontal cortex of AD patients [267, 268] and in post-synaptosomal supernatants derived from AD brain [269]. Active GSK-3 also appears in neurones before the development of NFTs and it co-localises with dystrophic neurites and NFTs in later stages of the disease [269–271]. GSK-3 is also upregulated in peripheral lymphocytes in MCI and AD [272]. The importance of GSK-3 in the pathogenesis of AD has been emphasised by reports that a *GSK3B* polymorphism is a significant risk factor for the development of LOAD [273]. Both isoforms of GSK-3 (GSK-3β and GSK-3α) appear to induce the hyperphosphorylation of tau [274, 275], but GSK-3α alone regulates the cleavage of APP and would appear to exert this role in the very early phase of the disease [276–278]. However, increased GSK-3β signalling also seems to play a pathological role in amyloid processing as such signalling increases *BACE1* expression, thereby facilitating the increased production of Aβ [279]. Conversely, and unsurprisingly, inhibition of this enzyme leads to a reduction in Aβ production [279]. There is evidence that GSK-3α enhances the activity of the γ-secretase complex [277] and may act to downregulate the activity of α-secretase [280]. There is also accumulating evidence, albeit in vitro, demonstrating that ApoE4 increases *GSK3B* expression, potentially leading to the exacerbation of pathology associated with the activation and upregulation of this kinase [281, 282].

## Oxidative Stress mTOR Activation and Impaired Autophagy and UPS Clearance

### Background

Autophagy encompasses a series of pathways by which damaged cytosolic components are transferred to lysosomes and subjected to enzymatic degradation in an immunologically silent manner (reviewed in [283]). There are three recognised subgroups of autophagy, namely macroautophagy, the dominant form in human cells, microautophagy and chaperone-mediated autophagy. Readers interested in a detailed examination of these processes and the differences and similarities between them are referred to a comprehensive review by Yu and others [284]. The UPS, on the other hand, is based on the receipt of ubiquitin-tagged oxidatively damaged and/or misfolded proteins by the barrel-shaped 26S proteasome, composed of multiple protein subunits, via a narrow opening (see [285]). Once ensconced, such proteins are subjected to a range of proteolytic enzymes, ultimately producing ubiquitin-tagged monomers [286].

The autophagic process is upregulated in the brains of AD patients, most notably in the hippocampus and other areas of the brain associated with AD pathology [287]. These observations may be significant in terms of differentiating AD from normal ageing, as there is copious evidence that the autophagic process is downregulated in normal ageing [288, 289]. However, despite the transcriptional upregulation of autophagy seen in AD patients, the weight of evidence indicates that autophagic lysosomal clearance is dysregulated and defective in the hippocampus of AD patients, even in those in the very early stages of their disease [287] [24].

The activity of the UPS is also impaired in the hippocampus and other disease-susceptible brain regions in AD patients, but apparently not in brain regions not associated with AD-specific neurodegenerative pathology (reviewed by [285, 286]). It would also appear that changes in the protein composition of the 26S proteasome and impaired activity of ubiquitin C-terminal hydrolase L1 (UCH-L1), a deubiquitinating enzyme responsible for the production of ubiquitin-tagged monomers, may be a characteristic of AD [290, 291]. From the perspective of this paper, it is especially noteworthy that downregulation of this enzyme appears to be the result of oxidative damage and occurs in AD patients many years before any evidence of amyloid plaques or NFTs [286, 291].

### Oxidative Stress and mTOR Activity in AD

mTOR is recognised as one of the master regulators of cellular metabolism in general and in autophagic processes in particular [292]. Readers interested in the many homeostatic roles, biochemistry and mechanistic actions of the two mTORC (mTOR complex) isoforms are referred to excellent reviews by Laplante and Sabatini [293, 294].

The weight of evidence suggests that mTOR activity is increased in the temporal cortex and hippocampus of AD patients [295, 296], and an activated but dysregulated Akt/mTOR signalling pathway in the hippocampus would appear to be a universal feature of AD and MCI (reviewed by [297]). It is noteworthy that *MTOR* expression is normally increasingly inhibited in the ageing brain [298, 299], and hence the existence of elevated mTOR activity in the hippocampus of AD patients could be a factor underpinning dysfunctional autophagic lysosomal clearance in that region of the brain, as discussed above. From the wider perspective of AD pathology, mTOR has several roles, such as the regulation of many aspects of synaptic function and protein aggregation, and is known to promote ptau and tau dyshomeostasis [300–303]. Some authors also propose that intricate molecular interactions between Aβ, tau and mTOR exacerbate the rate of cognitive decline [304, 305]. Elevated mTOR signalling is also relevant from the perspective of the more ‘generic’ elements involved in disease pathogenesis as this kinase regulates mitochondrial function [306], immune cell homeostasis [307] and levels of oxidative stress [308]. It is also of interest that mTOR activity in AD does not appear to be modified by *APOE* allele status, which hints that this molecule could play a unique role in AD pathology which is not seen in normal ageing [300].

### Oxidative Stress and Compromised UPS Function and Structure

Initially, increased levels of oxidative stress provoke a defensive response whereby the 26S proteasome dissociates into its 20S and 19S subunits, with the former being resistant to oxidative damage and thus responsible for protein degradation in this changed environment [309–311]. This adaptive response has limitations however, and during the development of chronic oxidative stress, the 20S subunit as well as the 26S proteasome may also become deactivated [309, 312], leading to the accumulation of insoluble covalently crosslinked proteins which can further inhibit the proteasome [310, 313]. Proteasomal dysfunction can lead to decreased degradation of misfolded proteins, thus resulting in accumulation of oxidised proteins and subsequent protein aggregation. Protein aggregates can then feedback, further to inhibit proteasomal activities, generate additional cellular stress and lead to cytotoxicity [309, 310, 314]. Additionally, oxidatively modified proteins may impair the cellular machinery of autophagic degradation [314, 315]. Reactive species can damage the lysosomal membrane and crosslinked membrane proteins, resulting in cytosolic leakage of lysosomal hydrolases [315, 316].

**Fig. 3:**
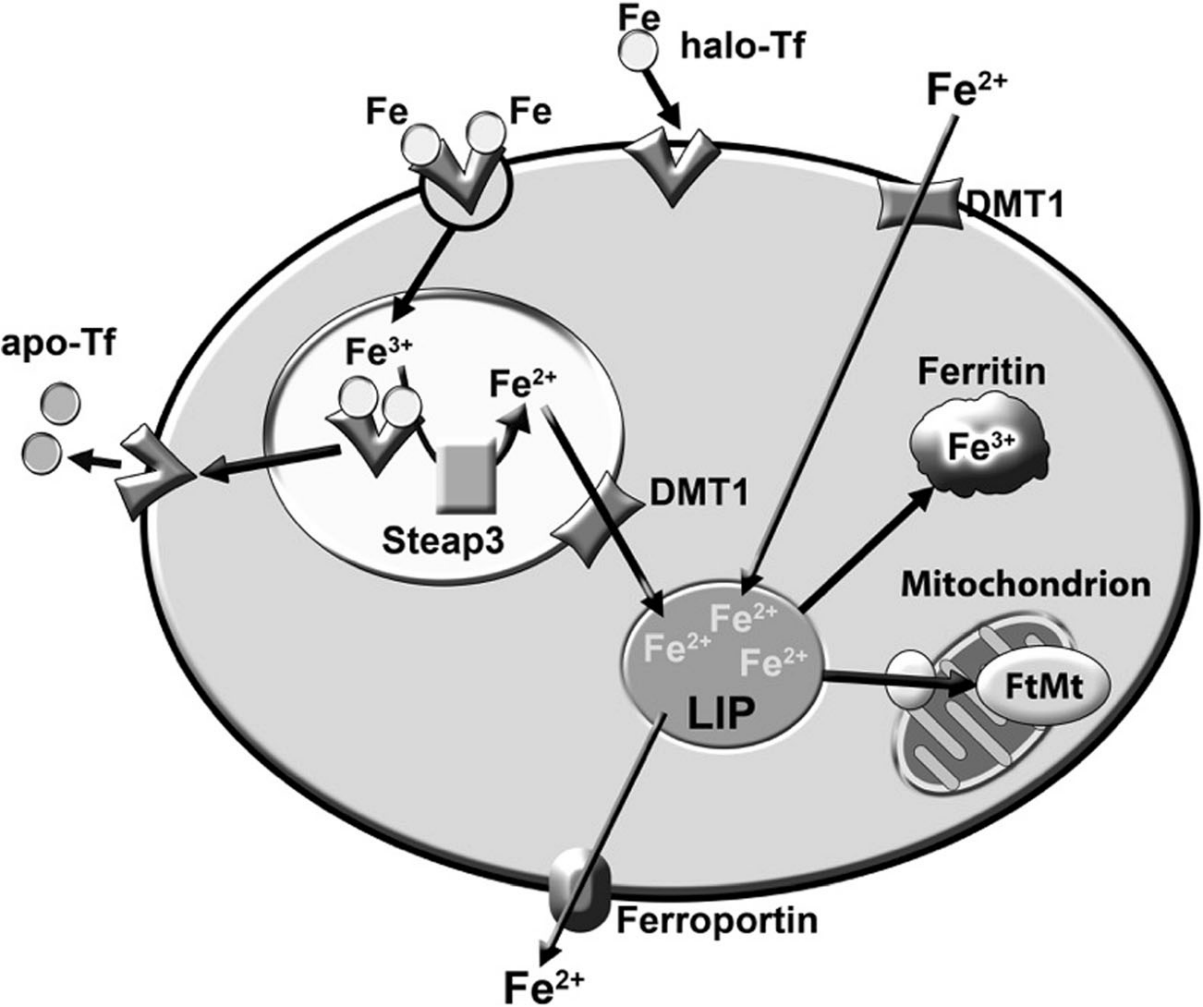
Iron homeostasis in neurones. Neurones and glial cells can uptake iron bound to transferrin (TBI), or bound to other molecules such as citrate and ATP secreted by astrocytes (NTBI). Neuronal uptake of TBI is enabled by the transferrin receptor located at the cell membrane and the uptake of NTBI in inflammatory conditions is probably enabled by DMT1. DMT1 and TfR1 complexes are internalised via endocytosis, ultimately resulting in the release of redox-active iron (Fe(II)) into the cytosol and the return of other molecules in the complexes to the plasma membrane. Once in the cytosol, Fe(II) can be utilised for various essential metabolic processes such as the synthesis of iron-sulphur proteins, or sequestrated by cytosolic ferritin and mitochondrial ferritin (FtMt), which offers protection against the advent of the Fenton reaction. Iron is removed from neurones by ferroportin, supported by the multi-copper-containing ferroxidase caeruloplasmin and sAPP, which both act to stabilise ferroportin at the cell surface

When considered as a whole, the data demonstrate that chronic oxidative stress impairs autophagy by provoking unfavourable changes in autophagic degradation, inhibition of lysosomal enzyme function and lysosomal membrane damage [317]. Furthermore, some oxidatively modified aggregated species are resistant to degradation by proteases and accumulate within lysosomes. There, the non-degraded proteins become a potential new source of reactive species, further damaging the lysosomal membrane [318]. This oxidative damage to lysosomal lipid membranes can be exacerbated by high levels of iron seen in AD patients, which increase the sensitivity of these membranes to oxidative damage to the point of inducing apoptotic or necrotic cell death resulting from lysosomal rupture and release of toxic hydroxylases, calpains and redox-active iron into the cytoplasm [139]. There is a growing appreciation that the role of redox-active iron and iron dyshomeostasis as a driver of neuropathology in AD may be pivotal in AD both as a source of increasing oxidative stress, via hydroxyl production through the Fenton reaction, and in the development of amyloid- and tau-related pathology. Hence, the role of oxidative stress in the development of iron dyshomeostasis and accumulation in the brains of AD patients and the pathological consequences of this phenomenon is the focus of the remainder of this paper. Understanding the content below requires some knowledge of the factors involved in maintaining iron homeostasis, which are depicted in Fig. 3 and summarised in the accompanying legend.

## Oxidative Stress and Iron Accumulation in AD

### Evidence of Iron Dyshomeostasis in AD Patients

Sophisticated MRI approaches have allowed the detection of increased iron levels in the brains of AD patients, most notably in the putamen and in posterior GM and white matter regions [319–321]. Elevated iron levels in the cortex and cerebellum are also a commonly reported phenomenon in MCI patients [322]. Levels of intracellular iron are subject to strict homeostatic regulation at the translational and transcriptional levels.

### Transcriptional Regulation of Iron Homeostasis

Regulation at the transcriptional level is mediated by interplay between the iron transport exporter protein ferroportin-1 (fpn-1) and the peptide hormone hepcidin, whereby increased activity of the latter leads to a reduction in the activity and levels of the former, hence reducing the cellular export of iron [323, 324]. Crucially, hepcidin synthesis is upregulated in an environment of chronic oxidative stress and neuroinflammation as a result of elevated H_2_O_2_ [325] and/or IL-6-activated STAT3 [326–329]. In fact, lower H_2_O_2_ concentrations (in the range of the levels observed during inflammation) require STAT3 phosphorylation to induce hepcidin and may, synergistically with IL-6, stimulate hepcidin [325]. This is clearly one mechanism underpinning the adverse effect of neuroinflammation and oxidative stress on iron accumulation in the CNS. Several authors have also reported that upregulation of divalent metal transporter 1 (DMT1) on the surface of neurones and glial cells results from the release of TNF-α, IL-1β, IL-6 and NO by LPS-activated microglia [330–332]. Importantly, the release of PICs from activated microglia, most notably IL-6, also leads to increases of hepcidin and reduction of ferroportin in neurones, which supplies a mechanism allowing increasing levels of neuronal iron accumulation over time in an environment of neuroinflammation [330, 331, 333].

### Regulation of Iron Homeostasis at the Translational Level

**Fig. 4:**
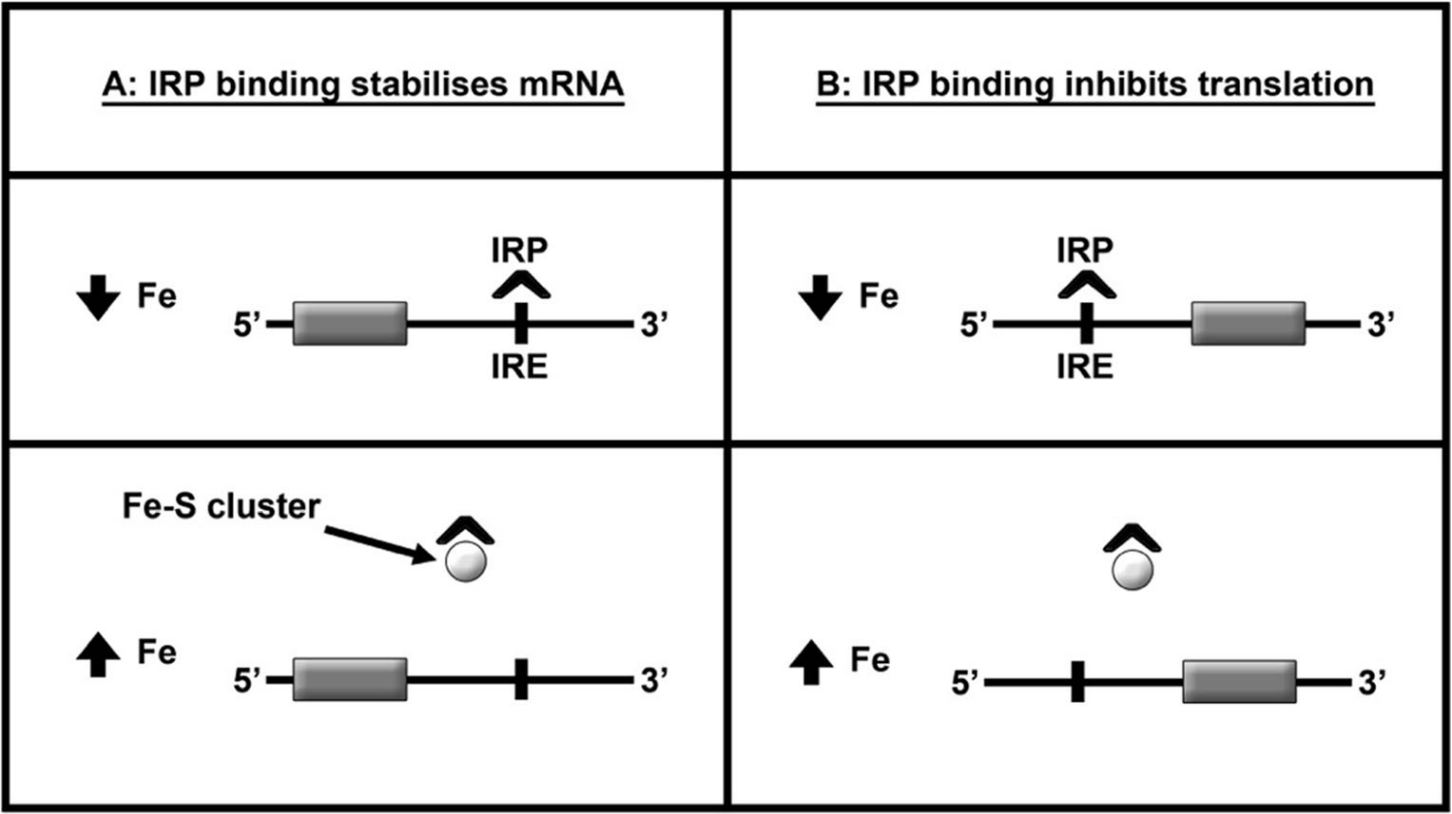
Post-transcriptional control of iron homeostasis in neurones and glial cells. Binding of IRP1 and IRP2 to IRE in the 5′-UTR of mRNAs encoding ferritin and ferroportin represses translation, while binding of IRP1 and IRP2 to IRE in the 3′-UTR of mRNAs encoding TfR1 and DMT1 stabilises the mRNA resulting in efficient translation. In an environment of increasing oxidative stress, IRP2 is degraded while IRP1-mRNA binding is enhanced, which inhibits the synthesis of ferritin, ferroportin and APP while simultaneously upregulating the production DMT1 and TfR1. The cumulative effect of these activities is significantly increased iron

Regulation of iron homeostasis and the translational level are governed by iron regulatory proteins (IRP) 1 and 2, which can bind to iron response elements (IRE) in the 5′ or 3′ untranslated regions (UTRs) of the mRNA sequences responsible for the production of proteins involved in iron homeostasis. This interplay is described as the IRP/IRE system (reviewed by [334]). The organisation and function of this system is depicted in Fig. 4 and explained in the accompanying legend.

### Detrimental Effect of Oxidative Stress on the IRP/IRE System

In an environment of increasing oxidative stress, IRP1 RNA binding is enhanced which inhibits the synthesis of ferritin, ferroportin and APP while concomitantly upregulating the production of DMT1 and transferrin receptor (TfR1) [335]. The cumulative effects of these activities are significantly increased iron uptake, a major reduction in iron sequestration and increased uptake of transferrin-bound iron (TBI) and non-transferrin-bound iron (NTBI) [335–339]. NO, and indeed peroxynitrite, also increase the mRNA binding of IRP1-IRE sequences [340, 341]. Elevated levels of NO also promote the degradation of IRP2 via a number of mechanisms including *S*-nitrosylation of crucial cysteine residues [342, 343], leaving IRP1 as the sole IRP regulating iron levels in brain cells in an environment of chronic oxidative and nitrosative stress. It is also interesting to note that IRP1-IRE complexes appear to be the only active complexes in the brains of AD patients [344]. In addition, it is noteworthy that recent findings indicate that increased APP activity and aggressive Aβ deposition seen in AD patients result, at least in part, from iron accumulation and dysfunctional IRP-IRE signalling [345, 346]. The role of iron accumulation in increased APP production is further highlighted by evidence demonstrating that iron chelation selectively downregulates *APP* mRNA production [347, 348].

### Effect of Elevated Fe(III) and Fe(II) on the Development of Amyloid and Tau Pathology

#### Effect on APP Processing

*APP* translation is regulated by IL-1 activity and the IRE element in the 5′ UTR of *APP* mRNA. This IRE region interacts with IRP1 in human brain cortical tissue [348, 349]. Therefore, increasing levels of iron can stimulate the translation of *APP* by provoking the dissociation of IRP1 as described above [348]. Hence, prolonged increases in neural iron have the effect of increasing the amount of APP available for amyloidogenic processing and Aβ production. In addition, elevated iron also reduces the α-secretase cleavage of APP and favours proteolysis by β-secretase [350, 351]. Mechanistically, this phenomenon stems from the capacity of iron to reduce the transcription of the proconvertase furin. Under physiological conditions, furin initiates cleavage of A dysintegrin and metalloproteases 10 (ADAM10) and TNF-α-converting enzyme (TACE) and increases the activity of α-secretase and hence the production of α-secretase-derived secreted form of APP (sAPPα) [352]. However, iron-induced suppression of furin transcription enhances the activity of β-secretase activity, thereby stimulating the amyloidogenic pathway and thus the production of Aβ_1–42_ [351, 353]. The plausibility of this mechanism in vivo is further reinforced by evidence demonstrating that furin levels are reduced in the brains of AD patients [352].

#### Effect of Elevated Fe(III) and Fe(II) on Amyloid Plaque Formation

There is some evidence to suggest that the initial seeding of Aβ_42_ plaques with Fe(III) may be beneficial by facilitating the export of excessive insoluble iron via microglial phagocytosis and subsequent lysosomal degradation [354]. However, several research teams have produced preclinical data demonstrating that prolonged association of Fe(III) with Aβ_1–42_ leads to the reduction of the former and increased levels of redox-active Fe(II) [355, 356]. These findings have been recently reproduced in vivo in cortical tissue of AD transgenic mice [357]. Furthermore, these authors reported a direct correlation between elevated Fe(II) levels resulting from the reduction of Fe(III) by Aβ_1–42_ and pathological changes in plaque morphology particularly with regard to the protein/fibril density of fibrillar fragments and diffuse plaques [357]. The formation of Fe(II)/Aβ complexes in AD patients is important from a pathological perspective as Fe(II) has the capacity to interact with Aβ amino acids, subsequently conferring longitudinal changes to the normal patterns of amyloid formations [358, 359]. In brief, the interactions between Fe(III) and Fe(II), on the one hand, and APP and Aβ, on the other hand, influence the speed and extent of Aβ aggregation into fibrillary structures [360, 361]. More specifically, the weight of evidence suggests that when enough amyloid deposition has occurred, toxic oligomeric formations can propagate in a nonlinear amyloidogenic positive feedback loop, bypassing the normal requirement for amyloid monomers to form dimers [362], thereby accelerating Aβ aggregation, oligomerisation and amyloidogenesis [363, 364]. The role of iron in this process may be of paramount importance as there is evidence to suggest that Aβ plaques may not be neurotoxic in the absence of iron and that the oxidative and peroxidative damage to proteins and lipids associated with Aβ stems from its high affinity with iron and its capacity to reduce Fe(III) to Fe(II) thereby providing a redox-active Aβ-iron complex capable of producing destructive hydroxyl radicals in association with elevated hydrogen peroxide produced by soluble Aβ_1–42_ [190] reviewed by [365]. The indispensable role of elevated Fe(II) and Fe(III) as drivers of amyloid pathology is further supported by evidence obtained from rodent models of AD demonstrating that iron chelation can prevent Aβ aggregation and reverse memory loss [363, 366].

#### Effect of Elevated Fe(III) and Fe(II) on Tau Hyperphosphorylation and NFT Formation

Fe(II) can induce tau hyperphosphorylation [367, 368] via a mechanism involving the activation of the MAPK pathway and the extracellular signal-regulated kinase 1/2 (Erk1/2) pathway [369, 370]. This effect may be inhibited in vivo via the use of the iron chelator deferoxamine [368, 371]. This may induce a cascade of self-amplifying pathology as the hyperphosphorylation of tau and the formation of NFTs in the brains of AD patients results in an increased production of haem oxygenase-1 (HO-1) [372, 373] which provokes the release of Fe(II) [374], which may exacerbate ROS production via Fenton chemistry [353, 375]. Increasing levels of ROS may also explain the elevations in cytosolic copper and zinc seen in AD patients, which would occur as a result of oxidation of binding-thiol groups in their sequestration partner metallothionein [376, 377]; reviewed by [378]. It is also noteworthy that high levels of oxidative stress in neurones would be expected to inhibit PP2A [30, 379] and such inhibition could well add to the neurotoxic milieu, as reduced PPA2 activity is associated with neuronal death via a mechanism involving the activation of MAPK [380]. The pathological role of PP2A inactivation in the neurones of advanced AD patients may well be underestimated as there is evidence suggesting that impaired signalling of this phosphatase is a major element underpinning the hyperphosphorylation of tau in Parkinsonian dementia, often described as ‘a classical tauopathy’ [381].

## The Function of the BBB in This Model

Several authors have reported reduced expression of adhesion molecules and tight junction proteins in BBB endothelial cells combined with a dysfunctional and/or disrupted neurovascular unit in AD patients with early disease long before the occurrence of dementia and in the absence of neurodegeneration and brain atrophy [382–384]. Importantly, such damage may result from the presence of prolonged systemic inflammation and elevated levels of PICs, which increase the permeability of tight junctions by decreasing levels of glycocalyx and other adhesion molecules, as well as causing endothelial cell damage and disruption of the of glia limitans [385, 386]. It is important to note that such damage may result from PICs in the systemic circulation or following activation of microglia and astrocytes in the brain and the latter phenomenon goes some way to explaining the dysfunction of the neurovascular unit seen in early AD patients described above [382, 385, 386].

## Other Neurodegenerative Disorders

This peripheral model may help explain other neurodegenerative disorders besides AD. Numerous in vivo studies have demonstrated a dysfunctional or disrupted BBB and neurovascular unit in other neurological diseases such as PD [387, 388]. This is of interest as a recent meta-analysis has confirmed the presence of peripheral inflammation and elevated PICs in PD patients [389]. Moreover, the development of BBB disruption and the subsequent egress of activated T cells and other lymphocytes into the CNS further exacerbating microglial activation have been established as a causative factor in the pathogenesis of the illness [390]. The ultimate cause of chronic peripheral inflammation in PD patients is not entirely understood and may be multifactorial. However, it seems reasonable to conclude that pesticide exposure and perhaps a history of head trauma may be involved, as both factors appear to play as a causative role in the development of the illness [391, 392].

## Conclusion

In conclusion, it has been shown that the presence of the *APOE* ε4 allele, and epigenetic dysregulation, including increased DNA methylation and altered miRNA expression, could explain increased levels of peripheral and central inflammation and oxidative stress in AD. Furthermore, this increased oxidative stress and inflammation could originate in the periphery rather than in the CNS itself. Finally, molecular neurobiological mechanisms have been adduced which explain how the initial development of elevated peripheral and central inflammation and oxidative stress in the context of genetic and epigenetic abnormalities could explain the development of AD.
